# When control isn’t controlled: How different experimental sham control conditions complicate TMS evidence in addiction

**DOI:** 10.1016/j.dadr.2026.100450

**Published:** 2026-05-23

**Authors:** Ghazaleh Soleimani, Zhihe Zhao, Malte R. Güth, Alexander Opitz

**Affiliations:** Department of Biomedical Engineering, University of Minnesota, MN, USA

**Keywords:** Sham condition, Transcranial magnetic stimulation, TMS, Addiction, Coil tilt

Appropriate control conditions are essential for interpreting outcomes in non-invasive brain stimulation studies, including transcranial magnetic stimulation (TMS). For example, out of 140 addiction-focused TMS trials conducted through the end of 2024 (Soleimani, Souki, et al., 2025), we identified substantial heterogeneity in the selection of control conditions (Fig. 1.a). The tilted-coil approach was the most commonly used method, despite evidence that the residual induced electric field (E-field) may remain sufficiently strong to produce biological effects (Lisanby et al., 2001, Loo et al., 2000). Findings from simulations of low-intensity TMS (Zhao et al., 2025) demonstrate that even subthreshold E-fields can modulate calcium signaling, neurotransmitter release, synaptic plasticity, neuroinflammatory pathways, and glial function, with measurable behavioral and clinical consequences (Moretti and Rodger, 2022).

**Fig. 1:**
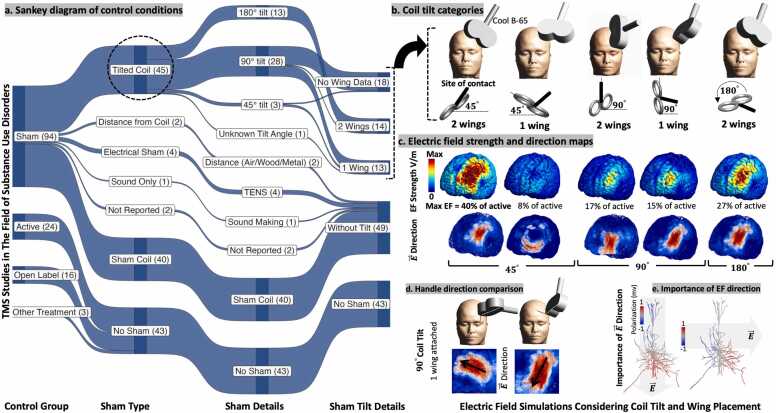
Control conditions used in TMS studies of substance use disorders and estimated electric fields under different sham configurations. (a) **Sankey diagram summarizing sham control conditions used across 140 TMS studies in substance use disorders.** This Sankey diagram illustrates the distribution of TMS studies in the field of substance use disorders based on their control-group design, sham type, specific sham implementation details, and characteristics of coil tilt and wing attachment. The width of the boxes in each column reflects the relative prevalence of each category, while the width of the connecting ribbons shows how studies flow from one category to the next (e.g., from “Sham” to specific tilt angles and wing-attachment configurations). “Tilted coil sham” represents studies that angled the coil to reduce active stimulation, which is the main focus here. Only 94 of 140 studies employed a sham-controlled design, while the remaining studies used active TMS controls (n = 24), open-label approaches (n = 16), or alternative treatments (n = 3). Even within sham-controlled studies, the methodology varied: two did not report details, 40 used sham coils, while others employed electrical stimulation, sound-only shams, or increased coil-to-scalp distance. Notably, 45 studies (half of sham-controlled studies) relied on a tilted-coil method, with further variability in tilt angle (45°, 90°, or 180°) and wing attachment to the scalp (one vs. two wings). (b) The definition of coil tilt and pad/wing contact varies across studies. Illustrations show the MagVenture Cool B-65 coil positioned at nominal 45°, 90°, and 180° tilt angles relative to the scalp. “Wing attachment” refers to whether one or both coil wings contact the scalp surface. (c) **Simulated cortical electric field strength** (first row) **and direction** (second row) under different tilt and wing-attachment configurations. **(d) Effect of coil handle orientation on electric field distribution.** Simulations of a 90°-tilted coil with one wing attached demonstrate that coil handle direction often unreported substantially alters the resulting electric field direction, despite identical tilt angle and wing configuration. **(e) Importance of electric field direction for neural polarization.** Fiber-orientation-based polarization maps illustrate how differences in electric field direction (not just magnitude) influence the predicted neuronal response, emphasizing that small variations in coil placement and orientation can lead to meaningful differences in cortical activation even in “sham” configurations.

Building on our established E-field modeling frameworks (Opitz et al., 2013, Opitz et al., 2011, Shirinpour et al., 2021, Thielscher et al., 2011) and our prior work applying individualized approaches to clinical TMS protocols (Soleimani et al., 2025, Soleimani et al., 2024, Soleimani et al., 2025), we quantified sham-induced fields under commonly used tilted-coil configurations. We expanded on this by simulating the sham TMS-induced E-field in a human head model (Opitz et al., 2011) to quantify how coil tilt, wing attachment, and coil handle orientation influence the E-field. The primary aim was to highlight the need for transparent reporting of sham methodology and the possibility that sham stimulation may induce measurable cortical E-fields, thereby influencing active–sham effect sizes.

Our results showed that active TMS delivered over F3 generated a peak E-field of 201.5 V/m in the left DLPFC. In contrast, sham TMS produced markedly lower—but still non-negligible—fields, ranging from 8% of the active (45° tilt, one wing in contact with the scalp) to 40% (45° tilt, two wings in contact). Consistent with prior reports (Loo et al., 2000, Opitz et al., 2015), E-fields of this magnitude may be sufficient to elicit motor responses when applied over the motor cortex and, in other cortical regions, may still induce meaningful neurobiological effects (Moretti and Rodger, 2022, Radman et al., 2009). For example, a concurrent TMS-EEG study have demonstrated that patterned stimulation protocols can induce measurable electrophysiological modulation even at stimulation intensities around 40% of resting motor threshold (Zmeykina et al., 2020). Currently available evidence does not support a universally accepted E-field threshold below which TMS can be considered biologically inert. Rather, available findings suggest that physiological effects may exist along a continuum and depend on multiple interacting factors, including stimulation pattern, target location, field orientation, neuronal morphology, brain state, and the sensitivity of the outcome measure. For instance, stimulation intensities that do not produce overt motor-evoked potentials may still influence cellular signaling, synaptic activity, or network-level electrophysiological dynamics. In substance use disorders specifically, additional factors such as substance type, duration of addiction, withdrawal state, and concurrent medications may further influence cortical excitability and susceptibility to stimulation effects.

Additionally, single-wing placements (45° or 90° tilt) produced fields predominantly oriented along a single axis (z or y, respectively), whereas two-wing placements resulted in a more evenly distributed field across components. Beyond variability in tilt angle and wing attachment, coil tilt itself lacks a standardized definition, and the induced cortical E-field is highly sensitive to coil handle orientation—a methodological detail not reported in any of the reviewed studies. Notably, even when tilt angle and wing attachment are held constant, altering handle direction alone can substantially change both the magnitude and orientation of the induced E-field (Fig. 1.d.). Because neuronal responses depend not only on E-field magnitude but also on field orientation relative to cellular morphology and axonal pathways (Fig. 1.e.), these directional differences may lead to distinct physiological effects even under sham conditions (Zhao et al., 2025).

Overall, sham TMS using tilted coils, illustrated here by addiction as a clinical application, may generate 8–40% of the active E-field and therefore reflect dose-comparison rather than true placebo designs. These findings underscore the need for greater methodological rigor in defining and evaluating sham conditions in clinical research. Specifically, we suggest: (1) distinguish between “placebo” controls and neurobiological active low-dose stimulation when tilted-coil shams induce measurable cortical fields; (2) report full sham parameters—including coil model, tilt angle, wing attachment, coil handle direction, coil-to-scalp distance, and, when possible, estimated E-field magnitude; (3) incorporate E-field simulations for both active and sham conditions to quantify biological plausibility rather than assuming inertness; (4) consider both magnitude and orientation of induced fields, given their relevance for neuronal and glial activation; and (5) reinterpret past sham-controlled trials with figure-of-eight tilted coils in addiction as dose-comparison designs when sham conditions likely induced low-intensity neuromodulatory effects. (6) To detect significant clinical or physiological differences, larger sample sizes may be required because biologically active sham conditions can artificially reduce active-versus-sham effect sizes. (7) Active-control designs may be a useful complement to conventional sham approaches in some contexts, as sham-related effects can arise not only from residual cortical E-fields but also from concurrent auditory and somatosensory stimulation. Standardizing sham methodology with these criteria will improve reproducibility, clarify effect size interpretation, strengthen statistical power considerations, and strengthen both clinical and mechanistic inferences in TMS research for substance use disorders.

## CRediT authorship contribution statement

**Ghazaleh Soleimani:** Writing – review & editing, Writing – original draft, Visualization, Project administration, Methodology, Investigation, Formal analysis, Data curation, Conceptualization. **Alexander Opitz:** Writing – review & editing, Validation, Supervision, Resources, Funding acquisition. **Malte R Güth:** Writing – review & editing. **Zhihe Zhao:** Visualization.

## Declaration of Competing Interest

The authors declare that they have no known competing financial interests or personal relationships that could have appeared to influence the work reported in this paper.
